# Bacterial Community Composition and Its Relationship with Environmental Factors in the Artificial Reef Area for Marine Ranching in Changhai County

**DOI:** 10.3390/ani15050639

**Published:** 2025-02-22

**Authors:** Jiamin Yan, Xu Wei, Liwei Si, Zheng Zhang, Jingsi Zhao, Liyu Deng, Tao Tian, Qingxia Li, Zengqiang Yin, Zhongxin Wu

**Affiliations:** 1 Center for Marine Ranching Engineering Science Research of Liaoning, Dalian Ocean University, Dalian 116023, China; 13332291130@163.com (J.Y.); m19818973602@163.com (X.W.); 19818932720@163.com (L.S.); zz13654291368@163.com (Z.Z.); dengliyudeyouxiang@163.com (L.D.); liqingxia@dlou.edu.cn (Q.L.); zqyin@dlou.edu.cn (Z.Y.); wuzhongxin@dlou.edu.cn (Z.W.); 2 College of Marine Science and Environment Engineering, Dalian Ocean University, Dalian 116023, China; 3 College of Fisheries and Life Science, Dalian Ocean University, Dalian 116023, China; 4 Industry Research Institute of Marine Ranching, Dalian Ocean University, Dalian 116023, China

**Keywords:** bacterial community, 16S rDNA, marine ranching, artificial reefs, North Yellow Sea

## Abstract

In this study, samples were collected from different types of artificial fish reefs and prevention and control areas in the sea areas of the northern part of Da Changshan Island and the northeastern part of Xiao Changshan Island in the North Yellow Sea. The purpose is to compare the differences in the bacterial communities among different regions, determine the impacts of environmental factors on the bacterial communities, and evaluate the ecological effects of artificial fish reefs on the marine bacterial communities. We obtained a total of 2,128,186 effective sequences and 4321 bacterial operational taxonomic units (OTUs), which were classified into 14 phyla and 76 genera. Proteobacteria were the most abundant phylum across the 32 samples, followed by Bacteroidetes. We found that all samples from the deep-sea control area exhibited the highest bacterial richness. In addition, all samples from the shallow-water concrete reef exhibited high community richness. The distribution of bacterial communities showed differences among different regions. In two specific sea areas, the bacteria in the sediment samples exhibited particularly remarkable characteristics of high diversity. Importantly, environmental factors significantly influence bacterial communities. In seawater samples, salinity (Sal) and dissolved oxygen (DO) were the primary factors affecting bacterial communities. Furthermore, grain size (GS) emerged as the most critical physicochemical factor influencing bacterial communities in sediment. This study compared the characteristics of bacterial communities in different types of artificial reefs and control areas in two marine ranches and revealed the main environmental factors affecting the bacterial communities. This is of great significance for protecting biodiversity and evaluating the ecological effects of artificial reef placement.

## Simple Summary

Artificial reef construction is a fundamental ecological project in marine ranching and a key method for creating habitats for marine life. Bacteria are important producers and decomposers in marine ecosystems and play a major role in marine biogeochemical cycles. Herein, we collected sediment and water samples from the artificial reef areas where two types of materials, natural stone and concrete, were used. In this study, we profiled the variations of bacterial communities among areas and materials in the water and sediment of artificial reefs using 16S rRNA gene sequencing.

## 1. Introduction

Marine ranching is a fishery model that creates or restores habitats required by marine organisms to reproduce, grow, feed, and avoid predators. Further, this model increases and conserves fishery resources, improves the ecological environments of marine areas, and allows for sustainable use of fishery resources through artificial reefs, stock enhancement and release, as well as other measures in a specific area [1,2,3]. Climate change is negatively impacting many marine fish stocks and fisheries, and many experts believe that marine ranching is an important solution for restoring fisheries [4,5,6]. Artificial reefs are man-made structures placed in aquatic environments to provide habitats or shelter for organisms. These structures have long been used to attract fish, and their development has intensified over the last three decades [7,8]. Artificial reefs are constructed using simple and widely used materials, including timber, natural stone, concrete, derelict boats, and oyster reefs [9,10,11,12]. Artificial reefs are critical tools in marine ranching, and their application has restored and improved marine ecological environments, increased and improved fishery resources, and promoted sustainable and healthy marine economic development [13,14].

Currently, basic research on artificial reefs is focused on artificial reef materials, reef structure, and hydrodynamic characteristics, evaluation of the ecological effects of artificial reefs, and monitoring and management of artificial reefs [15,16,17]. Every surface of solids in seawater will be covered by a biofilm, and the same is true for the artificial reefs placed on the seabed. A biofilm is a complex community composed of microorganisms and substances such as extracellular polymers secreted by them, which contains a variety of organisms [18,19,20]. Marine microorganisms are important constituents of marine ecosystems, participating in marine material cycles, energy flow, and maintaining marine ecosystem diversity and stability [21]. Most marine bacteria are decomposers, but some are producers and indicators of changes in marine conditions. This makes marine bacteria a vital part of the marine ecosystem, where they play a crucial role in the energy flow and biogeochemical cycling of different elements, including carbon, nitrogen, and sulfur, among others, as well as in the evolution of life on Earth [22,23,24]. Understanding the community composition and change patterns of marine bacteria will help us understand the structure of the microbial community and the mechanisms of interactions in artificial reef areas. Changhai County is located in the eastern part of Dalian City, and its marine aquaculture industry is highly developed. However, with decades of ocean fishing, pollution, and large-scale aquaculture, among other factors, the environmental and fishery resources in the seas and oceans of Changhai County have been severely damaged, and marine resources have been significantly depleted. To restore the marine environment and ensure the sustainable development of the fishing industry, Changhai County has actively implemented ecological restoration, seabed modification, artificial reef construction, biological bottom seeding, and other marine ranching strategies [25]. Previous studies have shown that the construction of artificial reefs in Changhai County waters can help improve the marine ecological environment and promote sustainable marine fisheries [26]. There is a close correlation between the material of artificial reefs and the microbial communities. Hala F et al. found that compared to traditional material artificial reefs, the microbial community structure on the surfaces of environmentally friendly artificial biological reefs containing bioactive materials underwent significant changes, with the richness and diversity of certain microbial communities on bioactive material-based reefs significantly increased [27]. Guo et al., in their study on concrete and wooden artificial reefs, discovered that the OTU richness and Shannon index of microbial communities on concrete artificial reefs were significantly higher than those on wooden reefs [28]. Therefore, we conducted a study on the bacterial communities of artificial reefs made of different materials in the marine ranch of Changhai County.

Due to their porous structures, chemical compositions, and attachment substrates, concrete artificial reefs and stone reefs may support higher microbial diversity and abundance. Therefore, we propose a hypothesis that the distribution of bacterial communities shows differences among different sampling areas. Key environmental factors such as temperature (T), dissolved oxygen (DO), salinity (Sal), and sediment grain size (GS) will significantly affect the structure and diversity of the bacterial community on artificial reefs. In the current study, the main objectives of the study were (1) to compare the differences in the microbial community structures on artificial reefs of different materials; (2) to analyze the impact of sea areas at different depths on the bacterial community; and (3) to identify the key environmental factors driving the dynamic changes of the microbial community.

## 2. Materials and Methods

### 2.1. Sample Collection

**Figure 1:**
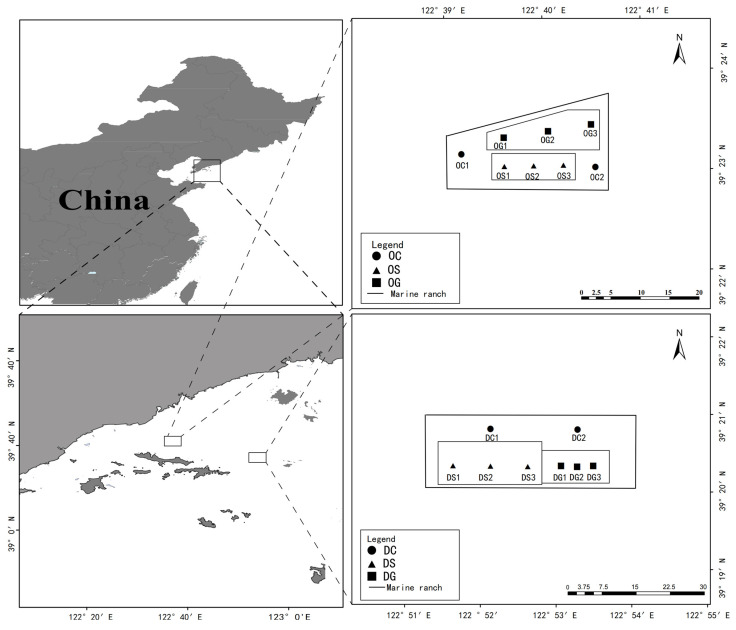
Sampling station map in May 2023 (OCn: Shallow water control area; OSn: Shallow water stone reefs; OGn: Shallow water concrete component reefs; DCn: Deep water control area; DSn: Deep water stone reefs; DGn: Deep water concrete component reefs).

This study was conducted in two marine ranching demonstration areas, including the deep-sea area of Xiao Changshan Island, Dalian City, and the artificial reefs in the waters of Xiao Changshan Island, which consist mainly of stone reefs and concrete component reefs that cover an area of 77.34 hectares. The shallow coastal waters are a national-level marine ranching demonstration site located in the sea area of Dalian Da Changshan Island. Stone reefs and porous cubic frame ecological reefs (2 m × 2 m × 2 m) with a single reef volume of 8 m^3^ have been constructed in the sea area of Dalian Da Changshan Island. Figure 1 shows a schematic diagram of the constructed 166 hectares of artificial reefs. Water and sediment sampling in the artificial reefs of Changhai County was conducted in early May 2023. The average water depths in the deep and shallow water areas were 26 m and 15 m, respectively. Three sites were established for stone AR areas and concrete artificial reefs, and two sites were established in control areas in both seas. For the water samples, we used a plexiglass container to collect 2 L of water at each site. For the sediment samples, a grab sediment sampler was applied to collect approximately 1 kg of surface sediment (0–10 cm) for analysis. Water and sediment samples were stored in a cooler filled with ice and immediately transported back to the laboratory within 2 h. In the laboratory environment, for the 2 L seawater samples collected at each site, the filtration technique was rapidly applied to obtain filter membranes. The extracted samples were carefully transferred into 50-mL sterile centrifuge tubes and then stored in a low-temperature environment of −80 °C for subsequent high-throughput sequencing analysis. The collection of surface sediment samples was completed using a grab sampler, and approximately 1 kg of sediment was collected at each site. Subsequently, 2–3 g was weighed from each sediment sample and transferred into 50-mL sterile centrifuge tubes, while the remaining sediment was properly placed in sterile bags, awaiting further processing procedures. The sediment placed in sterile bags was stored in a refrigerator at −20 °C for the determination of sediment physicochemical properties, and the samples stored in centrifuge tubes were stored in a refrigerator at −80 °C, specifically for high-throughput sequencing experiments. The methods for treating samples were followed as previously described by Fang et al. (2021) [29]. All field experiments were permitted by the Measures for Annual Evaluation and Reexamination of National Marine Ranching Demonstration Areas, which were endorsed by the Ministry of Agriculture and Rural Areas of China. The sampling included sediment and seawater samples. A total of 32 samples were collected in both deep water and shallow water areas, consisting of 16 water samples and 16 mud samples, covering 8 environmental factors. Deep artificial reefs with concrete elements, artificial reefs with stone elements, and control area sites are referred to as deep water control area (DCn), deep water stone reefs (DSn), and deep-water concrete component reefs (DGn), respectively. Shallow water concrete artificial reefs, stone artificial reefs, and control area sites are referred to as shallow water control area (OCn), shallow water stone reefs (OSn), and shallow water concrete component reefs (OGn), respectively.

### 2.2. Environmental Factor Measurements

Water samples were measured in situ for temperature, DO, pH, and Sal using a YSI PRO DSS multi-parameter water quality analyzer (YSI, Yellow Springs, OH, USA). All field experiments were permitted by the Measures for Annual Evaluation and Reexamination of National Marine Ranching Demonstration Areas. Analyze the total organic carbon (TOC) by the potassium dichromate oxidation-reduction volumetric method according to Part 5 of the Marine Monitoring Specification GB 17378.5-2007 (18.1). Analyze the total phosphorus (TP) by the spectrophotometric method according to Part 5 of the Marine Monitoring Specification GB 17378.5-2007 (Appendix C). Analyze the total nitrogen (TN) by the Kjeldahl titration method according to Part 5 of the Marine Monitoring Specification GB 17378.5-2007 (Appendix D). Analyze the sediment particle size according to Part 8 of the Specification for Oceanographic Survey GB/T 12763.8-2007 (6.3).

### 2.3. DNA Extraction and PCR Amplification

Total community genomic DNA extraction was performed using an E.Z.N.A™ MagBind Soil DNA Kit (Omega, M5635-02, Norcross, GA, USA). We measured the DNA concentration using a Qubit 4.0 (Thermo, Waltham, MA, USA) to ensure that adequate amounts of high-quality genomic DNA had been extracted. Our target was the V3–V4 hypervariable region of the bacterial 16S rRNA gene. PCR was initiated immediately after DNA extraction. The 16S rRNA V3–V4 amplicon was amplified using 2×Hieff^®^ Robust PCR Master Mix (Yeasen, 10105ES03, Shanghai, China). Two universal bacterial 16S rRNA gene amplicon PCR primers (PAGE purified) were used: the amplicon PCR forward primer (CCTACGGGNGGCWGCAG) and the amplicon PCR reverse primer (GACTACHVGGGTATCTAATCC). The reaction was set up as follows: microbial DNA (10 ng/µL) 2 µL; amplicon PCR forward primer (10 µM) 1 µL; amplicon PCR reverse primer (10 µM) 1 µL; 2×Hieff^®^ Robust PCR Master Mix (Yeasen, 10105ES03, China) to a final volume of 30 µL. The plate was sealed, and PCR was performed in a thermal cycler (Applied Biosystems 9700, Waltham, MA, USA) using the following program: 1 cycle of denaturation at 95 °C for 3 min, followed by 5 cycles of denaturation at 95 °C for 30 s, annealing at 45 °C for 30 s, elongation at 72 °C for 30 s, then 20 cycles of denaturation at 95 °C for 30 s, annealing at 55 °C for 30 s, elongation at 72 °C for 30 s, and a final extension at 72 °C for 5 min. The PCR products were checked using electrophoresis in 2% (*w*/*v*) agarose gels in TBE buffer (Tris, boric acid, EDTA) stained with ethidium bromide (EB) and visualized under UV light.

We used Hieff NGS™ DNA Selection Beads (Yeasen, 10105ES03, China) to purify the free primers and primer dimer species in the amplicon product. Samples were delivered to Sangon BioTech (Shanghai, China) for library construction using universal Illumina adaptors and indexes. Before sequencing, the DNA concentration of each PCR product was determined using a Qubit^®^ 4.0 Green double-stranded DNA assay, and quality control was performed using a bioanalyzer (Agilent 2100, Santa Clara, CA, USA). Depending on coverage needs, all libraries were pooled for one run. The amplicons from each reaction mixture were pooled in equimolar ratios based on their concentration. Sequencing was performed using the Illumina MiSeq system (Illumina MiSeq, San Diego, CA, USA) according to the manufacturer’s instructions. After sequencing, the two short Illumina reads were assembled using PEAR software (version 0.9.8) according to the overlap, and fastq files were processed to generate individual fasta and qual files, which were then analyzed by standard methods. The effective tags were clustered into operational taxonomic units (OTUs) of ≥97% similarity using Usearch software (version 11.0.667). Chimeric sequences and singleton OTUs were removed, and the remaining sequences were then sorted into each sample based on their OTUs.

### 2.4. Statistical Analyses

Microbial community diversity analyses, including both α- and β-diversity, were carried out in R (version 3.6.0) using the vegan package (version 2.5-6). Alpha diversity indices, including Chao1, Shannon, ACE, Simpson, Pielou’s, and sequencing depth (Good’s coverage), were calculated using mothur (https://www.ncbi.nlm.nih.gov/pmc/articles/PMC2786419/) (accessed in 15 June 2023) [30]. Differences in sediment microbial community alpha-diversity indices among groups were analyzed using Tukey’s test. Diversity analysis was mainly performed using R software (version 3.6.0) and associated data visualization packages. The Venn diagram package in R (UpsetR) was used to generate Venn diagrams (https://www.ncbi.nlm.nih.gov/pmc/articles/pmid/21269502/, https://www.ncbi.nlm.nih.gov/pmc/articles/pmid/28645171/) (accessed in 15 June 2023) [31,32]. Principal component analysis (PCOA) was performed using the WGCNA, stats, and ggplot2 packages in R. Analysis of similarity (ANOSIM) was conducted using the ANOSIM function in the R vegan package. Histograms showing the relative abundance of dominant bacterial taxa at the phylum and genus levels were generated using R. Covariance plots illustrating the relationships between microbial communities and environmental factors were also generated using R. Additionally, redundancy analysis (RDA) was primarily used to examine the relationship between community-based distances and environmental factors. The psych package (version 2.0.9) was utilized for these analyses. Specifically, for the calculation of α-diversity metrics of each sample, QIIME 2 was employed. These α-diversity metrics encompassed the number of observed species, the Shannon index, the Chao1 index, and Good’s coverage.

In a Pearson correlation analysis between bacterial communities and environmental factors, Spearman correlation coefficients between genera or species and environmental factors were calculated and tested for significance using the corr.test function in the psych package. The results were then visualized using the pheatmap function in R.

## 3. Results

### 3.1. Changes in Physicochemical Characteristics of Overlying Water and Sediment

Thirty-two samples were divided into six groups according to the sample properties and sampling areas (Table 1). DO levels at deep-water sites were significantly higher than those at shallow-water sites. Specifically, the DO values at the DG and DS sites reached 9.63 mg/L and 9.64 mg/L, respectively, representing the highest DO levels among all the measured sites. In contrast, the temperature at deep-water area sites was lower than that at shallow-water area sites. Regarding salinity and pH, their values were significantly higher in deeper waters. For salinity, the values at deep-water sites ranged from 30.61‰ to 30.76‰, while at shallow-water sites, they were in the range of 29.69‰ to 29.86‰. The pH values also showed a similar trend, with higher values in deep waters (8.21–8.31) compared to shallow waters (8.05–8.16). The TOC in the control area is higher than that in the artificial reef area, while the TP is lower than that in the artificial reef area.

The particle size analysis of surface sediments indicated clear differences between deep and shallow waters. In deep waters, sand (S: 63–2000 μm) was the dominant grain group. All samples had sand contents greater than 76.7%, and the mean median grain sizes ranged from 137.08 to 157.53 μm. In shallow waters, sandy chalk (ST: 4–2000 μm) was the dominant grain group, with all samples having contents above 80.0%. The mean median grain sizes in shallow waters ranged from 19.36 to 32.20 μm. Additionally, the median particle size in DGR and OG was significantly smaller than that in DS and OS. Moreover, the median particle size in OG was smaller compared to that in OS and OC (Table 1).

### 3.2. Bacterial Diversity Analysis

**Figure 2:**
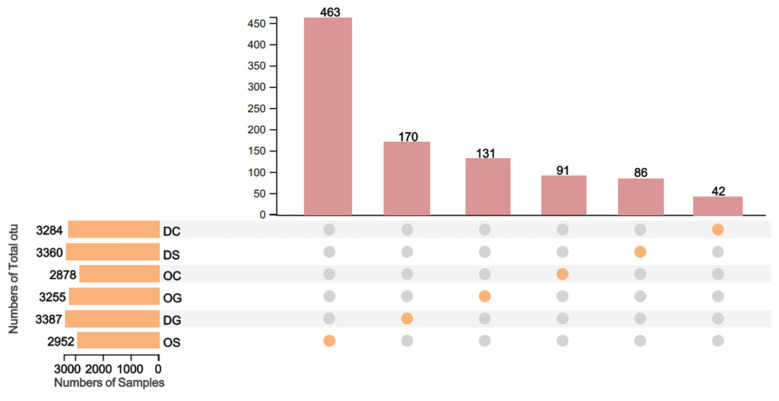
The number of fungal OTUs specific to sample intersections. The left panel displays the number of OTUs in each sample. Samples with shared OTUs are connected by dots and lines in the dot matrix area. The bar chart in the top panel shows the number of shared OTUs at the intersections represented by the connecting dots.

Using 16S rRNA high-throughput sequencing technology, a total of 2,195,261 raw sequences were obtained from 32 samples. After splicing, quality control, and filtering out chimeric sequences, 2,128,186 valid sequences were retained. The non-redundant sequences from each sample were extracted and clustered into OTUs based on 97% similarity (excluding singletons). During the clustering process, chimeric sequences were removed, resulting in 4321 OTUs. There were 463 OTUs that only appeared in the OS sample and were absent in the other samples. The number of unique OTUs specific to DG, OG, OC, DS, and DC was 170, 131, 91, 86, and 42, respectively (Figure 2).

**Figure 3:**
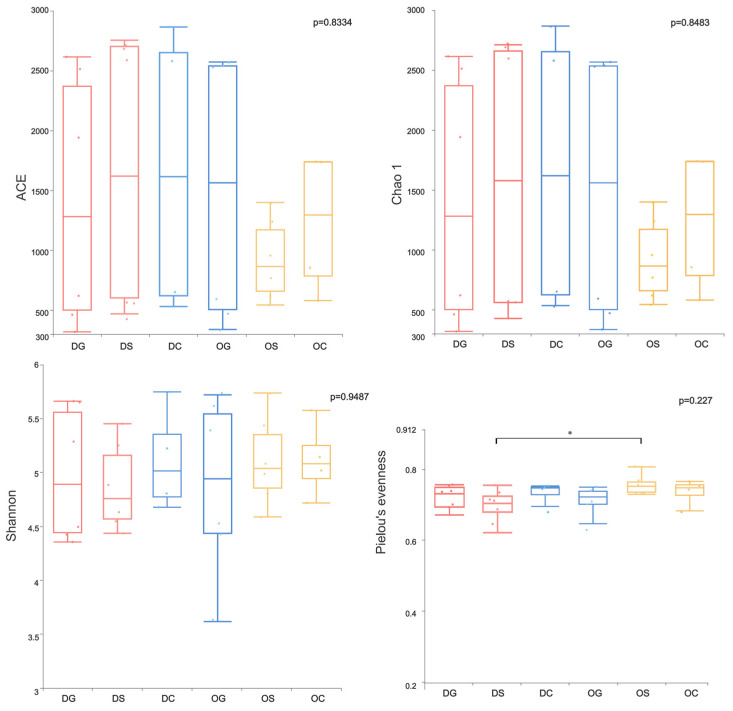
Four alpha diversity indices (ACE, Chao1, Shannon, and Pielou’s evenness) among the six groups. * Indicates that there are significant differences between the DS and OS groups.

Figure 3 shows the four alpha-diversity indices (ACE, Chao1, Shannon, and Pielou’s evenness) among six groups, and a significance analysis of the differences among the six groups was performed. Among all sample groups, the DS group has the highest ACE and Chao1 indices, while the OG group has the lowest values for these two indices. The DG group has the highest Shannon index, and the Pielou’s evenness index is the highest in the OS group. In the deep-water sampling area, the two indices (ACE and Chao1) of the DS group are higher than those of the DC and DG groups, and the two indices (Shannon and Pielou’s evenness) of the DG group are higher than those of the DC and DS groups. In the shallow-water sampling area, the three indices (ACE, Chao1, and Shannon) of the OG group are higher than those of the OC and OS groups, and the Pielou’s evenness index of the OS group is higher than those of the OC and OG groups.

**Figure 4:**
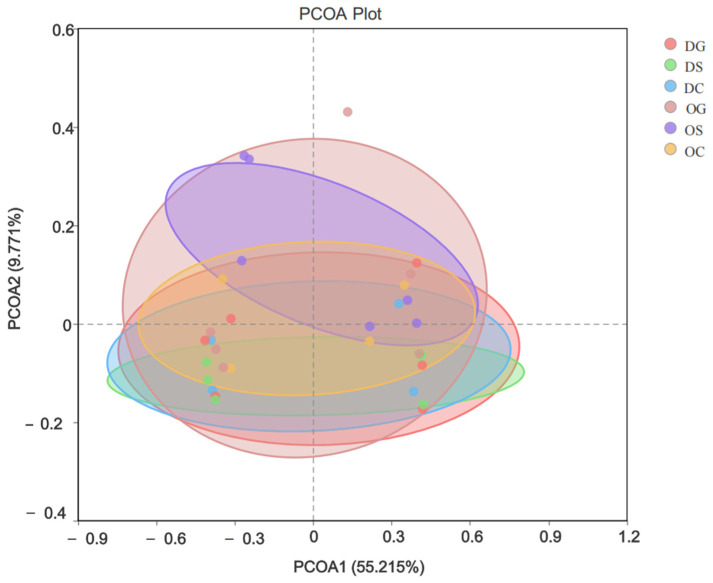
Principal component analysis based on the six groups.

The PCOA results (Figure 4) showed that the horizontal and vertical principal components contributed 55.215% and 9.771%, respectively, to explaining the group differences. Samples within the deep-water region and those within the shallow-water region exhibit a high level of internal similarity. The pronounced clustering of samples from the concrete artificial reef zone, the stone reef zone, and the control zone strongly indicates a high degree of similarity in their community compositions. Meanwhile, there are certain differences in the bacterial communities between the stone reef zone and the concrete reef zone. A portion of the samples from OS and OG is relatively scattered compared to those from other areas, suggesting that the similarity between the shallow-water stone reef zone and the shallow-water concrete reef zone is rather low and that there exist some variations in sample compositions.

ANOSIM is a non-parametric test used to determine differences between groups. Our ANOSIM data (Table 2) show a negative R-value in the five control groups: control area sediment-stone reef sediment, control area sediment-component reef sediment, stone reef seawater-control area seawater, stone reef seawater-component reef seawater, and component reef seawater-control area seawater. These data suggest that the differences between these five groups are smaller than the differences within the groups. Except for these five groups, the R-values for the bacterial community structure were positive, indicating that the differences between groups were greater than the differences within groups. At the same time, the P values for the CS-CW, CS-GW, SW-CS, SW-SS, SW-GS, SS-CW, SS-GS, GS-CW, and GW-GS groups were less than 0.05, indicating significant differences in the structure of the bacterial communities between the groups. Moreover, the P values for the CS-SS, CS-GS, SW-CW, SW-GW, and GW-CW groups were greater than 0.05, suggesting that there were no significant differences in the bacterial community within the group.

### 3.3. Compositional and Structural Analysis of Bacterial Communities

**Figure 5:**
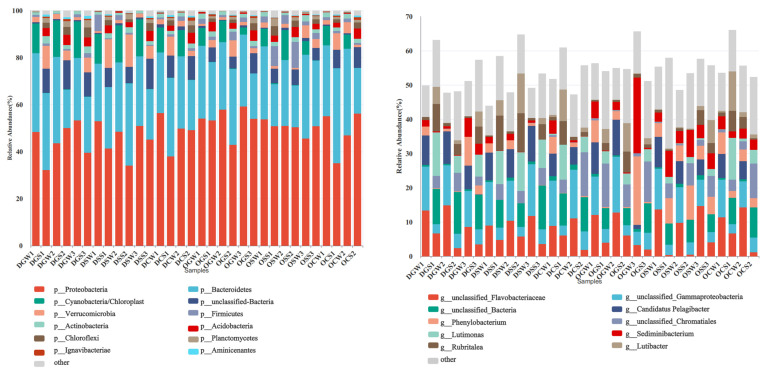
Relative abundance plot of bacterial communities at the gate level. Relative abundance plot of bacterial communities at the genus level. (In this figure, samples ending with “W” represent seawater samples, while those ending with “S” represent sediment samples.).

The bacterial community composition at the phylum level in the artificial reef area and the control area is shown in Figure 5. A total of 13 bacterial phyla were detected from the 32 analyzed samples. Proteobacteria was the most abundant phylum in the northern Yellow Sea waters and among all samples collected at the phylum level. Furthermore, Proteobacteria was the most abundant phylum identified in different samples from the GR, SR, and CR in both waters, with a relative abundance of 32.18–59.22%, followed by Bacteroidetes (16.33–40.45%). In comparison, Cyanobacteria had a low relative abundance in sediment samples, and its relative abundance in seawater samples ranged from 2.96 to 15.72%. Additionally, the relative abundance of Verrucomicrobia was higher in deep-water samples than in shallow-water samples, while Firmicutes had the highest abundance (1.06–8.53%) in the stone reef area in shallow water. The relative abundance of Actinobacteria and Acidobacteria did not differ significantly between reef samples of different materials in different waters.

At the genus level, 76 bacterial genera were detected in 32 samples. We plotted a bar chart of the community composition for the top ten dominant species. The dominant genera changed according to the sampling area, with unclassified Flavobacteriaceae and unclassified Gammaproteobacteria being the absolute dominant genera in deep and shallow waters. The relative abundance of Phenylobacterium was higher on artificial reefs in shallow waters than in deep waters, ranging from 0.23% to 20.08%, and Luteimonas was more abundant in the shallow-water control area. The relative abundance of Candidatus Pelagibacter and Luteimonas was higher in the deep-water artificial reefs than in control areas. In addition, unclassified Chromatiales have a higher abundance in shallow-water areas than in deep-water areas.

### 3.4. Relationship Between Bacterial Communities and Environmental Factors

**Figure 6:**
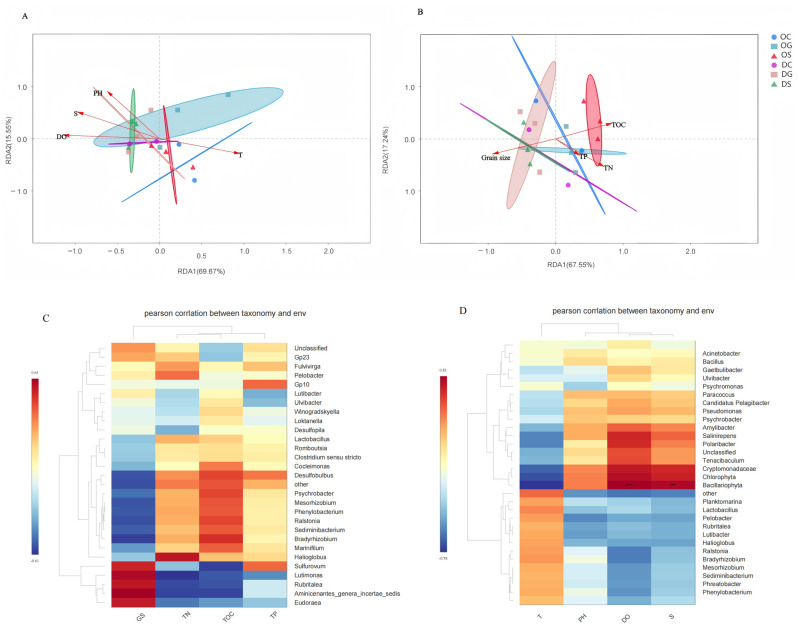
(**A**) Redundancy analysis diagram of water environment factors. (**B**) Redundancy analysis diagram of sediment environmental factors. (**C**) Heatmap of correlation between bacterial community at genus level and sediment environmental factors. (**D**) Heatmap of correlation between bacterial community at the genus level and water environmental factors.

The relationship between bacterial communities and environmental physicochemical factors was explored using Redundancy Analysis (RDA) to analyze seawater physicochemical factors and sediment environmental factors. Further exploration of the relationship between microbial communities and environmental factors (Figure 6A,B) revealed that RDA1 and RDA2 accounted for 69.67% and 15.55% of the cumulative variance in the relationship between environmental factors and bacterial communities, respectively. Additionally, pH, salinity, and dissolved oxygen (DO) showed positive correlations, while temperature exhibited a negative correlation with these three parameters. Temperature and DO were identified as the primary factors influencing bacterial community structures in the seawater samples from the two marine regions. Importantly, these findings are consistent with those from a previous study on the characteristics of microbial community structures in a typical artificial reef in Weihai. TP, TN, and TOC showed positive correlations, while GS exhibited negative correlations with these three parameters. RDA1 and RDA2 accounted for 67.55% and 17.24% of the cumulative variance in the relationship between environmental factors and bacterial communities, respectively (Figure 6B). Moreover, the most critical physicochemical factor influencing bacterial community structure in the sediment samples from the two seas was found to be the mean particle size, followed by TN and TOC. To investigate the correlation between environmental factors and microorganisms, the top 32 genera in relative abundance in seawater samples and the top 29 genera in relative abundance in sediment samples were selected for Pearson correlation analyses with environmental factors (Figure 6C,D). Bacillariophyta showed significant correlations with DO and salinity.

## 4. Discussion

### 4.1. Marine Ranching Environmental Factors

We found that the salinity of GS, DO, and pH values in the samples from the deep-water area were significantly higher compared with those in the samples from the shallow-water area, while the TOC, TN, and TP in the samples from the shallow-water area were higher. The TP in the samples from the concrete component reef area was significantly higher than that in the samples from the control area and the stone reef area, which is consistent with previous studies [33,34,35]. The TOC in the artificial reef area was significantly lower than that in the control area (Fukunaga et al., [36,37,38]), and the sediment particle size in the AR area in shallow waters was smaller compared to the control area. We found that in the samples from the deep-water area, the particle size in the control area was smaller than that in the artificial fish reef area, which is inconsistent with the findings of Guo regarding the microbial community structure characteristics of the typical AR area in Weihai. This discrepancy may be related to the selection of the CR in deep water. Our results indicate that the particle size of concrete components in the artificial reefs was smaller compared to the rocky reef area, which can alter the bottom particle size of the sea area. In this study, we did not conduct seasonal surveys, which should be addressed in future research.

### 4.2. Characteristics of Bacterial Community Composition

At present, there are few studies on the structure and diversity of bacterial communities in the northern Yellow Sea. Bai Jie et al. (2009) investigated the distribution characteristics of marine bacterial communities at different sites in the northern Yellow Sea [39]. They compared seawater and sediments from coastal and offshore areas of the northern Yellow Sea and found that Proteobacteria was the dominant phylum at each site, which is consistent with our findings. The number of OTUs in the control area was higher than that in the artificial reefs, suggesting greater species richness in the control area. However, the OTUs in the artificial reefs in shallow waters were higher than those in the control area, which aligns with the high bacterial abundance and diversity in the AR area reported by Yu et al. [40]. The richness of bacterial communities in the control area within deep waters was the highest, while the bacterial abundance in concrete component reefs in the shallow water area was significantly higher compared to the other two sampling areas. The construction of artificial reefs has led to the formation of specific community compositions and increased the abundance of dominant species [29,41,42,43]. Through diversity analysis, it was found that the bacterial diversity and the number of OTUs in the artificial reef area were higher than those in the control area, which is consistent with the hypothesis we put forward. In this study, only the composition of bacterial communities was investigated, while fungal communities were not examined. Future research should include the analysis of multiple microbial communities. Yao Jia [44] previously proposed that there are few differences in community composition and function between metal and cement biofilm microbiomes, but oil pollution would increase the relative abundance of biofilm bacteria in artificial reefs [45,46,47]. However, another study showed that oil contamination reduced the relative abundance of Proteobacteria in cement biofilms. Hauke F. Kegler et al. (2017) studied the bacterial community composition of coral reef habitats along a transcontinental shelf environment and depth gradient, revealing significant differences in bacterial community composition between nearshore water and sediment samples [48]. Depth has an important impact on bacterial community composition. However, we did not account for the depth effect at each site, only the average depth of the two sea areas, and the influence of oil pollution was not considered. Therefore, the impacts of depth and oil pollution on bacterial community structure should be investigated in future studies.

### 4.3. Main Drivers of Bacterial Community Structure Changes in Marine Environments

Li et al. (2013) have previously proposed that the structure and composition of dominant bacterial communities vary with environmental changes and that nutrient content, species, temperature, tide, flow field, and salinity in the environment may affect their distribution and species composition. As an important marine fishery resource and aquaculture organism, oysters have the ecological functions of purifying water bodies, creating habitats, and sequestering carbon [49,50,51]. In this work, we found that bacterial community composition in the concrete artificial reefs, the stone reef area, and the control area was similar. Importantly, we also found that the bacterial diversity and abundance of the stone reef area were slightly lower compared to the control area. This may be related to the impact of aquaculture activities on water quality, such as the release of certain substances during the farming process that can alter the water environment and thus affect the bacterial community. Some scholars have studied the environmental factors affecting planktonic bacteria in the aquaculture areas of the East China Sea and found that in summer and autumn, chemical oxygen demand, salinity, dissolved oxygen, nitrite nitrogen, etc. [52] all have an impact on the bacterial community. Previous studies by some scholars have shown that the abundance and diversity indices of the bacterial community exhibit obvious seasonal trends. During the course of this research investigation, we have not investigated the seasonal factors [53,54]. In order to explore microbial diversity more deeply and thoroughly, it is necessary for us to systematically conduct seasonal surveys in our subsequent research work.

In our investigation, we found that Sal and DO have a significant impact on the bacterial community. During sample collection and analysis in this study, we found that there was a significant difference in temperature between the two sea areas. Moreover, because of the different depths, sampling in the deep-water area was carried out in the morning and in the shallow-water area in the afternoon, respectively. Our data showed that TP and TN were slightly higher in the shallow waters, which may be due to the fact that the shallow waters are closer to the continental shelf and are, therefore, more affected by human and aquaculture activities, as well as sewage discharge. In this study, sediment particle size analyses in the two sea areas showed that the particle size of the concrete artificial reefs was smaller than that of the stone artificial reefs. Furthermore, we showed that particle sizes in the control area in the deep-water area were smaller compared to those of the artificial reefs. This may be because the mud collector is susceptible to the influence of ocean currents during the sampling process, and there may have been errors in the specific sampling sites, which would affect the distribution of sediment particle size.

A diverse range of factors can influence changes in microbial community structure, including the geographical properties of sedimentary materials, physicochemical properties (T, DO, Sal, pH, current, nutrients, heavy metals, etc.), food webs, and human activities, among other factors [55,56,57,58]. Among the eight environmental factors measured in this study, TN, DO, and temperature were identified as the main factors driving dynamic changes in bacterial community structures at the OTU level.

## 5. Conclusions

In conclusion, this study evaluated two different types of artificial reefs and the control areas in the waters of Dalian Da Changshan Island and Xiao Changshan Island using 16S rRNA sequencing technology. We analyzed the bacteria in both seawater and sediments, explored the differences in the bacterial community structures in water and sediments, and linked our findings to the characteristics of different habitats. The structures of bacterial communities and the compositions of dominant groups in different habitats vary due to environmental factors. The application of artificial reefs has improved the ecological environment of the surrounding sea areas and enhanced the diversity and stability of the bacterial community structures in these areas. In this study, we also revealed that the community structures and diversities of bacteria in artificial reefs are influenced by different physical and chemical factors, habitats, and materials. Judging from these research results, the positive impacts of artificial reefs on the marine ecological environment are multi-faceted. It is not only reflected in biodiversity but may also affect the material cycling and energy flow of the entire marine ecosystem by changing the bacterial community structures. This also suggests that in future marine ecological restoration and fishery resource management, we can plan the placement of artificial reefs more scientifically. According to the environmental characteristics of different sea areas, we can select appropriate reef materials and placement methods to maximize ecological and economic benefits. At the same time, with the continuous development of technology, more advanced technical means, such as real-time monitoring technology, may be introduced to more accurately capture the dynamic processes of the changes in bacterial community structures over time and with environmental changes.

## Figures and Tables

**Table 1:** Environmental factor data for deep- and shallow-water seas.

Groups	T (°C)	DO (mg/L)	Sal (‰)	pH	TOC (mg/L)	TP (mg/g)	TN (mg/g)	GS (μm)
DG	10.17 ± 0.21	9.63 ± 0.05	30.63 ± 0.11	8.31 ± 0.16	0.86 ± 0.03	0.49 ± 0.03	1.42 ± 0.39	153.33 ± 8.11
DS	10.3 ± 0.10	9.64 ± 0.07	30.76 ± 0.18	8.22 ± 0.10	0.85 ± 0.07	0.46 ± 0.07	1.81 ± 0.12	157.53 ± 2.57
DC	10.3 ± 0.14	9.47 ± 0.25	30.61 ± 0.35	8.21 ± 0.08	0.92 ± 0.12	0.41 ± 0.08	1.98 ± 0.42	137.08 ± 8.45
OG	12.3 ± 0.10	9.18 ± 0.06	29.86 ± 0.12	8.16 ± 0.10	1.14 ± 0.07	0.50 ± 0.02	2.10 ± 0.05	19.36 ± 4.41
OS	12.45 ± 0.25	9.29 ± 0.05	29.78 ± 0.09	8.05 ± 0.01	1.15 ± 0.06	0.48 ± 0.01	2.03 ± 0.14	20.11 ± 2.03
OC	12.35 ± 0.07	9.21 ± 0.03	29.69 ± 0.11	8.08 ± 0.06	1.23 ± 0.04	0.46 ± 0.11	1.99 ± 0.10	32.20 ± 2.80

(DG: Deep-water concrete elements for artificial reefs; DS: Deep-water stone artificial reefs; DC: Deep-water control zone; OG: Shallow water concrete component artificial reefs; OS: Shallow water stone artificial reefs; OC: Shallow water control zone).

**Table 2:** Analysis of similarities.

Groups	Sample Size	R	*p*	Permutation Number
CS-CW	8	1.000	0.026	999
CS-SS	10	−0.131	0.829	999
CS-GS	10	−0.162	0.885	999
CS-GW	10	1.000	0.009	999
SW-CS	10	1.000	0.007	999
SW-CW	10	−0.012	0.400	999
SW-SS	12	0.996	0.002	999
SW-GS	12	1.000	0.005	999
SW-GW	12	−0.061	0.784	999
SS-CW	10	0.992	0.007	999
SS-GW	12	0.015	0.350	999
SS-GS	12	0.963	0.006	999
GS-CW	10	1.000	0.005	999
GW-CW	10	−0.004	0.422	999
GW-GS	12	0.996	0.006	999

(CW: Seawater samples from the control area; CS: Sediment samples from the control area; GW: Seawater samples from the concrete component reef area; GS: Sediment samples from the concrete component reef area; SW: Seawater samples from the stone reef area; SS: Sediment samples from the stone reef area.).

## Data Availability

The original contributions presented in this study are included in the article. Further inquiries can be directed to the corresponding author.
